# Parents and Early Life Environment Affect Behavioral Development of Laying Hen Chickens

**DOI:** 10.1371/journal.pone.0090577

**Published:** 2014-03-06

**Authors:** Elske N. de Haas, J. Elizabeth Bolhuis, Bas Kemp, Ton G. G. Groothuis, T. Bas Rodenburg

**Affiliations:** 1 Adaptation Physiology Group, Department of Animal Science, Wageningen University and Research, Wageningen, The Netherlands; 2 Behavioural Biology, Centre for Behaviour and Neuroscience, University of Groningen, Groningen, The Netherlands; 3 Behavioural Ecology Group, Department of Animal Sciences, Wageningen University and Research, Wageningen, The Netherlands; CSIRO, Australia

## Abstract

Severe feather pecking (SFP) in commercial laying hens is a maladaptive behavior which is associated with anxiety traits. Many experimental studies have shown that stress in the parents can affect anxiety in the offspring, but until now these effects have been neglected in addressing the problem of SFP in commercially kept laying hens. We therefore studied whether parental stock (PS) affected the development of SFP and anxiety in their offspring. We used flocks from a brown and white genetic hybrid because genetic background can affect SFP and anxiety. As SFP can also be influenced by housing conditions on the rearing farm, we included effects of housing system and litter availability in the analysis. Forty-seven rearing flocks, originating from ten PS flocks were followed. Behavioral and physiological parameters related to anxiety and SFP were studied in the PS at 40 weeks of age and in the rearing flocks at one, five, ten and fifteen weeks of age. We found that PS had an effect on SFP at one week of age and on anxiety at one and five weeks of age. In the white hybrid, but not in the brown hybrid, high levels of maternal corticosterone, maternal feather damage and maternal whole-blood serotonin levels showed positive relations with offsprings’ SFP at one week and offsprings’ anxiety at one and five weeks of age. Disruption and limitation of litter supply at an early age on the rearing farms increased SFP, feather damage and fearfulness. These effects were most prominent in the brown hybrid. It appeared that hens from a brown hybrid are more affected by environmental conditions, while hens from a white hybrid were more strongly affected by parental effects. These results are important for designing measures to prevent the development of SFP, which may require a different approach in brown and white flocks.

## Introduction

In mammals, but also in avian and fish species, mothers can affect the behavioral development of their offspring both before and after birth or hatch (e.g. humans [1], [2], rodents [3], [4], fish [5], wild birds [6] and domesticated birds [7]; for reviews see: [8]–[10], farm animals [11], birds [12], [13]). Mechanisms by which birds may pass information to their offspring are through hormone transfer to the egg [12], [14] and/or via epigenetic pathways [15]–[17]. By these mechanisms the developing embryo may be better prepared for its future environment; this is also referred to as a “predictive adaptive response” [18], [19]. In poultry, yolk-hormone levels can vary according to stressful environmental conditions [20]. Exposure to repeated, unpredictable events (Japanese quail [21], domestic chicken [16]) and daily exposure to humans (Japanese quail [22]) can alter egg-hormone levels. Stress experienced by the hen can also reduce her own body weight [7] and egg weight [23], [24], and in this way influence offspring development too. Such maternal effects may underlie the repeated finding that offspring of stressed birds have higher anxiety levels compared with offspring from non-stressed birds [7], [21], [25]–[27].

These maternal effects may have important implications for the poultry industry, but have so far been overlooked. In commercial laying hens, feather pecking (FP), the plucking of- and pecking at feathers of conspecifics [28], is a maladaptive behavior. The severe form of FP (severe feather pecking: SFP) has serious consequences for animal welfare as it causes pain and stress in the recipient and can lead to mortality due to cannibalism. Counter measures against FP, such as beak trimming, adjustments of light intensity or supply of foraging materials [29], are only partially successful and we studied the possibility that maternal effects play a role. The tendency to develop SFP seems to be related to anxiety-related behavioral and physiological traits [30]–[33]. For example, chicks which show high anxiety in an Open Field test (social isolation in a novel environment) have stronger tendencies to perform SFP [30], [31], [33], [34]. Also, birds with high anxiety levels show high post-stress plasma corticosterone levels whilst having low whole-blood serotonin levels, which were linked to feather pecking tendencies [32], [33]. The predisposition to be more anxious and develop FP has a genetic component, as birds of a white ancestor origin are generally more anxious than birds of a brown origin [24], [34]–[38]. The predisposition for anxiety can be affected by level of stress of the parents [7], [39]. Therefore, it is important to assess this relationship under commercial conditions where it can affect millions of laying hens. In the poultry industry, parental flocks (parent stock: PS) are flocks which contain thousands of breeder hens and roosters housed together. They produce a multitude of offspring flocks (rearing flocks) which themselves contain thousands per flock. Additionally, the housing conditions during the offspring’s early life can affect development of behavior [40], [41] including FP [42], [43]. Factors such as a large group size [44], [45], a high stocking density [46], [47] and a lack of litter or unsuitable litter [48]–[50] have been shown to increase the development of FP.

In this study, we examined in two crosses of laying hens (Dekalb White: DW and ISA Borwn: ISA) whether parent stock had an effect on the development of FP and anxiety in their offspring. To understand the relation between parents and offspring, we studied which behavioral and physiological parameters (feather damage, plasma corticosterone levels and serotonin levels) of the parent stock coincided with high levels of SFP and anxiety in their offspring. In addition, we studied how litter supply and housing conditions during rearing affected the development of FP. Commercial PS flocks had an impact on the development of anxiety and SFP in their offspring, especially for the DW hybrid. Litter conditions and housing system also showed to have a substantial effect on SFP and anxiety, especially for the ISA hybrid.

## Materials and Methods

As one-on-one relations between parents and offspring cannot be determined under commercial conditions - due to the impossibility of individual recognition within large flocks of birds - data were assessed on flock level for both PS and rearing flocks.

### Ethics Statement

This study comprises an on-farm longitudinal follow-up study on commercial laying hens, conducted between August 2010 and March 2012, which was approved by the Institutional Animal Care and Use Committee of Wageningen University, The Netherlands (permit number for parental flocks: DEC 2010042, permit number for rearing flocks: DEC 2010083).

### Parent stock

#### Experimental animals and housing

Ten commercial flocks of parent stock (PS) of the rearing company Ter Heerdt BV, Babberich, The Netherlands were studied. Five of these were ISA Brown (ISA) parent stock (white hens, brown roosters) and five were Dekalb White (DW) parent stock (white hens and roosters). ISA Brown PS chickens originate from a Rhode Island Red and a Rhode Island White founder line. Dekalb White chickens originate from two White Leghorn founder lines. The ten PS flocks were situated at 7 different breeding farms, meaning that 3 farms had both hybrids while the remaining had either DW or ISA only. Flocks of different hybrids from the same breeding farm were taken as separate flocks. Rooster/hen ratio was approximate 1∶10 for all flocks. Flocks were kept on commercial propagator farms with floor housing, partly slatted floors, and litter. For details on housing see [24].

#### Measurements

At 40 weeks of age, levels of feather damage, basal plasma-corticosterone and whole-blood serotonin levels of parental hens were assessed. For a detailed description of the measurements, see [24]. For 20 hens per flock, blood samples were drawn from the wing vein within two min after capturing the hen. Blood samples were analyzed for plasma-corticosterone (CORT) and whole-blood serotonin (5-HT) levels (for details, see [24]). Each hen was individually taken from a random location in the chicken house (left or right; front or middle; floor or slats or nest boxes) to an adjacent room. After blood sampling, feather damage on neck, back and belly was assessed, and scored on a 3-point scale: no damage (a), slight damage (b), severe damage (c). Scores per area were summed to give a total body score [51] between 0 (no damage) and 2 (most severe damage). Fertilized eggs were collected daily and were incubated in a commercial incubator of the hatchery of Ter Heerdt BV, Zevenaar, The Netherlands. Fertilized eggs were collected per farm and hybrid. The pooled data per farm and hybrid are referred to as parent stock (PS).

### Rearing flocks

#### Experimental animals and housing

Per PS flock (n = 10) between three to seven rearing flocks were studied, of which 23 were DW and 24 were ISA (n = 47 rearing flocks in total). The 47 rearing flocks were situated at 25 different rearing farms. Age of the parents at time of incubation varied from 30 to 60 weeks of age, with a majority around 40 weeks. The rearing flocks contained only hen-chicks. At one day after hatch chicks arrived at the rearing farm on which they stayed until approximate 17 weeks of age. All rearing flocks were housed in a tier-system of which 39 flocks were housed in an aviary system and 8 flocks in a floor system to which levels were gradually added (level system). All systems provided tiers, a litter area, slatted area, perches, multiple nipple drinkers and feeding troughs at different levels but no nest boxes or outdoor area. During the first five weeks of life, in the aviary system adjacent cages were either closed, restricting the number of chicks within the same enclosure (between 30–60), or partly-open (between 30–100). Chicks in the level system were placed in one large flock which varied between 10.000 and 30.000 chicks. Upon arrival, chicks were housed under temperatures ranging between 30 and 33°C with humidity levels between 50 and 65%. Temperature was gradually decreased to approximately 19°C at 10 weeks of age, which was maintained from 10 to 17 weeks of age. Chicks were kept under artificial light either with or without additional LED light with intensities ranging from 1 – 25 LUX measured with a Voltcraft MS-1300 light meter (Conrad Electric Benelux, Oldenzaal, The Netherlands) on bird level. Light regime was a 4-h light/2-h dark cycle for the first seven days of life. After seven days, light regime was adjusted to a 16-h light/8-h dark cycle and light was subsequently decreased gradually from 16 to 9 consecutive hours per day. Each week, one hour of light was removed from the schedule, until 9 hours per day was reached (at 10 weeks of age). Chicks received a commercial diet: mashed starter 1 from one until four weeks of age; semi mashed starter 2 from four until ten weeks of age; and crumbled pre-lay diet from 10 until 17 weeks of age. Chicks were placed within the aviary system on cardboard paper (also called chick paper: [48]) varying from 50 to 90 grams per square meter. This cardboard paper prevented the chicks getting stuck or falling through the mesh wire of the system due to their small body size. It also enabled the accumulation of spilled food, excretions and/or litter and thus provided a foraging substrate. Around five weeks of age, exposure to the litter area within the system was enabled for all flocks. In the aviary system, all walls of the cage tiers were opened and the corridor between tiers became litter area. In the open level system the side walls of the system were opened, and the outside corridor became litter area. Litter supply could, however, be disrupted from seven to 10 days prior to opening the system by the removal of cardboard paper without additional litter being supplied (hereafter named litter disruption). Farmers use this approach to accustom chicks to their new flooring condition (i.e. wire or plastic surface without cardboard paper). Also, litter supply could be limited by supplying the cardboard paper remnants without additional flooring substrate such as wood-shavings or alfa-alfa (hereafter named litter limitation). The code of practice of maximum stocking densities was applied, enabling sufficient space per bird in the chicken house. Birds were vaccinated according to the standard vaccination protocol used by the rearing company. Extra specific vaccinations could be requested by the laying hen farm for which the birds were reared.

#### Measurements

**Figure 1:**
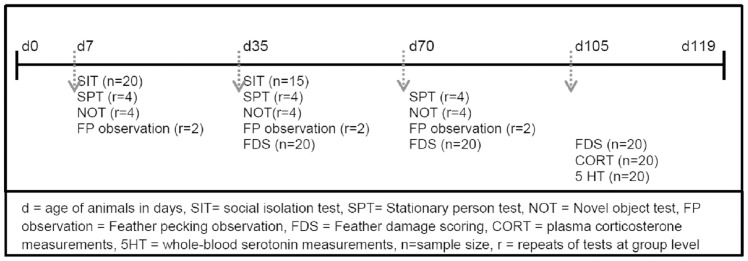
Time line of age of birds in days (d) with tests executed at specific ages.

At four age points during the rearing period behavioral observations were conducted: week one, five, ten and fifteen weeks of age (see Figure 1).

#### Anxiety related tests

Tests related to fear and anxiety were conducted at one, five and 10 weeks of age. Fear of humans was assessed by exposure to either a human arm in their home cage (at one and five weeks of age) or a human standing in the litter area (at 10 weeks of age). In the level system, fear of humans was assessed only by a human standing in the litter area at all ages. Fear of novelty was assessed by exposure to a novel wooden box (5*5*2 cm) with colored tape (red, yellow, white and green) at one and five weeks of age, and a novel stick with colored tape (a 50 cm PVC tube with colored tape) at 10 weeks of age [51]. In both tests, birds were exposed for two min to the human observer and the novel object separately. Every ten seconds, we counted the number of birds within close proximity (i.e. 25 cm). For the novel object test, we calculated at which time point at least three birds approached. As birds often did not approach within 25 cm during the human observer test, we estimated the minimal distance in cm of hens that approached over the total test duration. For each flock, tests were repeated four times at different locations in the chicken house (front, middle-front, middle-back, back) always under a light source to limit lack of visibility. A preliminary analysis was performed to assess the effect of location and as location did not affect the latency to approach the novel object or the minimal distance to the human observer, we averaged all values over our four tests. Separation anxiety was measured by a social isolation/novel environment test. Individual chicks, selected from random locations in the chicken house (n = 20 in week one, n = 15 in week five), were tested. Chicks were positioned inside a round orange bucket (30 cm Ø, with 30 cm height) at one week of age and a round white bucket (40 cm Ø, with 50 cm height) at five weeks of age for a duration of one min. At five weeks of age a larger bucket was needed to prevent chicks from jumping out the smaller bucket. The observer was out of sight of the chick while testing, but was able to record high pitched vocalizations; i.e. latency to vocalize and number of vocalizations. High pitched vocalizations are referred to as alarm or distress calls [52], [53]. They are interpreted as an attempt to reinstate contact with conspecifics and as indicating separation anxiety [54].

#### Feather pecking and feather damage

At one, five and 10 weeks of age feather pecking (FP) behavior was recorded during two 20-min observations in each flock. For each observation, FP was recorded by means of behavior sampling at a predetermined location of approximately 1 m^2^ within the chicken house, covering all resources (feeding through, drinking nipples, litter area, tiers and perches). FP was recorded as the frequency of pecks/20 min observation time. Gentle FP (GFP) was recorded as nibbling and gentle feather pecks without a reaction in the receiver, while severe FP (SFP) was recorded as forceful pecks with attempts to pull feathers out to back of the recipient body generally leading to a withdrawal response of the receiver [28], [42]. Aggressive pecks to neck and head, were also recorded but due to limited observation numbers, these data were not further analyzed. Prior to observations, the observer waited until birds were habituated to her presence by the criterion that 80% of chicks present were not directing their attention to the observer. The number of chicks within the observation area could vary between 15 and 50 chicks due to unrestricted physical boundaries. Feather damage was assessed at five, 10 and 15 weeks of age. At each age point, 20 chicks per flock, chosen selectively from random locations within the chicken house, were assessed for feather damage to the neck, back and belly region, similar to feather damage scoring in PS hens [51]. However, the wing and tail area were included as extra areas of measurement using a 0/1 scale, as slight damage to the tips of the feathers in these regions early in life possibly indicates the presence of SFP before severe damage is perceived. Total body score (FS) was the sum of values for all body regions, similar to the scoring system for PS hens, but damage to the tips of wings was added to the total body score as a value of 0.5.

#### Blood parameters

At 15 weeks of age, prior to assessment of feather damage, 20 hens per flock were blood sampled. Samples were always collected around 11–12 a.m. before feeding. An identical procedure was applied for blood sampling and analysis as with the PS hens (for details, see [24]). In short, individual hens were chosen selectively from random locations (floor, tier, perch, front and middle) in the chicken house and sampled within two minutes after capture. Blood (2.5-mL) was stored in 4-mL EDTA tubes and immediately put on ice. For whole-blood serotonin (5-HT) analysis, 1.1 mL of blood was pipetted out of the total amount and stored at −80°C. 1 mL of blood was used for analysis (see [32] for detailed description). 5-HT concentrations (nmol/mL) were assessed by fluorescence assay and compared with a standard curve of 5-HT stock of increasing dilutions. A Perkin-Elmer 2000 Fluorescence spectrophotometer was used to determine fluorescence at 283 and 540 nm. For basal plasma corticosterone (CORT) analysis, 1.4 mL of blood was centrifuged at 2,095 × g at 21°C for 6 min to obtain plasma. Plasma was stored at −20°C before CORT was analyzed at the Faculty of Bio Engineer Science, University of Leuven (Belgium). For the determination of corticosterone concentrations, a competitive radio-immunoassay was performed with the ImmuChem Double Antibody Corticosterone 125I RIA Kit for Rats and Mice of MP Biomedicals LLC (Bio-Connect Diagnostics BV, The Netherlands) with appropriately diluted plasma specimens (for details see [33]).

#### Statistical analysis

Data were analyzed with SAS 9.2. For each flock, flock averages were calculated. A general linear model (GLM) included the fixed effects of PS, hybrid (DW vs. ISA) and housing system (open, partly open, closed). For the variables which showed an effect of PS, an additional analysis was conducted to investigate the underlying factors. The average level of CORT, 5-HT and feather damage of the PS hens and age of the PS were added separately as a covariate in the model, which substituted the factor PS, and were tested with its interaction with factor hybrid. For the variables measured from five weeks of age onwards, the effects of limitation of litter (yes/no), disruption of litter supply (yes/no) and the interaction between limitation and disruption of litter supply, and their single interaction with hybrid were added to the model. Post-hoc least square means were used to assess pair-wise differences. Correlations between the residuals of the variables (based on a GLM with PS) were assessed, by hybrid, to determine relations between variables related to anxiety and FP. Plots were examined for outliers to confirm the calculated *R*-values. The normality of the distribution of the residuals was checked, and no transformations were needed. All data is expressed as means ± SEM.

## Results

### Parental effects

**Figure 2:**
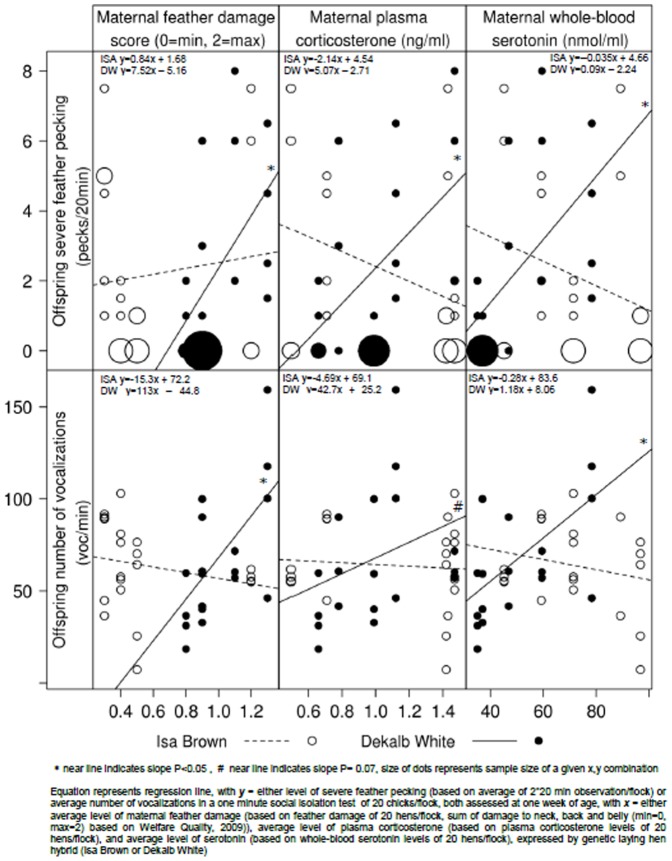
Average level of maternal feather damage [left panel], average level of maternal plasma-corticosterone [middle panel] and average level of whole-blood serotonin levels [right panel] with their offsprings’ average level of severe feather pecking at week one of age [upper panels] and the number of vocalizations in a social isolation at one week of age [lower panels].

SFP at one week of age was affected by parent stock (PS) (F_8,39_ = 4.09, P = 0.002). Additional analysis revealed that for the DW hybrid, but not for the ISA hybrid, offspring’ SFP at one week of age was related to high maternal plasma-CORT (CORT*hybrid: F_1,39_ = 6.25, P = 0.02), high maternal whole-blood 5-HT (5-HT*hybrid: F_1,39_ = 7.72, P = 0.01) and high maternal feather damage score (FS*hybrid: F_1,39_ = 5.02, P = 0.03), see Figure 2 [top panel]. For the ISA hybrid, no effects of maternal CORT, 5-HT or feather damage was found on offsprings’ SFP at one week of age. PS affected the number of vocalizations in the social isolation test at five weeks of age (F_8,43_ = 2.56, P = 0.03) and tended to affect the number of vocalizations at one week of age (F_8,39_ = 2.21, P = 0.06). PS did not affect the latency to vocalize at one week (F_8,39_ = 0.22, P = 0.98) or five weeks of age (F_8,43_ = 1.48, P = 0.20). Additional analysis revealed that for the DW hybrid but not for the ISA hybrid, a high level of vocalizations in the social isolation test at one week of age were related to high levels of maternal whole-blood 5-HT (5-HT*hybrid: F_1,39_ = 9.18, P = 0.005) and high maternal feather damage (FS*hybrid: F_1,39_ = 9.16, P = 0.005) and tended to relate to high levels of maternal plasma-CORT (CORT*hybrid: F_1,39_ = 3.48, P = 0.07) see Figure 2 [bottom panel]. High number of vocalizations at five weeks of age were related to high maternal feather damage in the DW hybrid (FS*hybrid: F_1,43_ = 5.98, P = 0.02: DW y = 38.4x – 26.98). For the ISA hybrid, no effects of maternal CORT, 5-HT or feather damage was found on number of vocalizations of the offspring at one or five week of age. Neither PS age nor its interaction with hybrid affected SFP, or vocalizations in the social isolation test at one week of age (SFP_week1_: PS age: F_1,39_ = 0.75, P = 0.39, PS age * hybrid F_1,39_ = 2.19, P = 0.15; vocalizations_week1_: PS age: F_1,39_ = 0.26, P = 0.61; PS age * hybrid F_1,39_ = 0.09, P = 0.76). PS did not affect SFP and GFP at five or ten weeks of age, feather damage, fearfulness at any other age.

### Housing effects

See table 1 for differences and pair-wise comparisons of housing system for FP, fear and feather damage. SFP at ten weeks and GFP at one and ten weeks was highest, and SFP at five weeks tended to be highest, in the open level system compared to the closed and partly-open aviary system (housing-system effect: SFP_week1_: F_2,39_ = 1.93, P = 0.16, SFP_week5_: F_2,43_ = 2.62, P = 0.10; SFP_week10_: F_2,45_ = 11.55, P = 0.002; GFP_week1_: F_2,38_ = 4.09, P = 0.03, GFP_week5_: F_2,44_ = 0.38, P = 0.69, GFP_week10_: F_2,45_ = 4.48, P = 0.02, see Table 1). Feather damage score at ten weeks, but not at five or fifteen weeks, was highest for flocks that were housed in an open level system compared to an aviary system (housing-system effect: FS_week5_: F_2,45_ = 1.81, P = 0.18, FS_week10_: F_2,45_ = 3.14, P = 0.05, FS_week15_: F_2,42_ = 1.26, P = 0.30). At one and five weeks of age, the latency of at least three birds to approach a novel object (NOT) was shortest in the open level system compared to the open and partly-open aviary system (housing-system effect: NOT_week1_: F_2,39_ = 17.02, P < 0.0001, NOT_week5_ F_2,45_ = 4.81, P = 0.01, NOT_week10_: F_2,43_ = 0.65, P = 0.53). In the fear for humans test at one week of age, the effect of housing-system was significant (F_2,39_ = 16.7, P < 0.0001: open: 96.1±26 cm, closed: 29±3.2 cm, partly-open: 23.6±1.8 cm). This effect is, however, an artifact caused by the different spatial dimensions of the systems on the test variable (minimal distance, i.e. the minimal distance can be larger in an open system vs. the other systems purely due to the systems’ spatial dimension) and the setting of the test (i.e. in the aviary systems response to a human arm, while in the level system response to a standing person is measured). Therefore, this results is not reported in Table 1. Housing system did not affect minimal distance to the human observer at five or ten weeks of age (housing-system effect: SPT_week5_: F_2,44_ = 0.13, P = 0.87; SPT_week10_:, F_2,44_ = 0.51, P = 0.60).

**Table 1:** Means ± SEM of response variables of the behavioral tests, feather pecking observations and feather damage scoring of rearing flocks housed in an open, closed or party-open system.

Variables				System		
Tests		Age	Response variables	Open (n = 8)	Closed (n = 25)	Partly open (n = 14)
***Stationary person test***						
	Week 1		Minimal distance (cm)	-	-	-
	Week 5		Minimal distance (cm)	71.7±19.2	78.2±13.7	74.7±22.9
	Week 10		Minimal distance (cm)	45.9±17.4	113.7±17.8	117.5±22.6
***Novel object test***						
	Week 1		Latency of 3 birds to approach (s)	**33.2±6.5^a^**	**87.6±6.0^b^**	**94.2±8.0^b^**
	Week 5		Latency of 3 birds to approach (s)	**17.2±2.6^a^**	**69.5±8.5^b^**	**68.1±11.5^b^**
	Week 10		Latency of 3 birds to approach (s)	14.3±1.7	30.0±5.6	24.8±4.6
***Social isolation test***						
	Week 1		Number of vocalizations/min	55.3±7.0	70.6±6.8	61.2±8.8
	Week 1		Latency to vocalise (s)	9.8±1.5	11.6±1.4	10.4±2.0
	Week 5		Number of vocalizations/min	24.5±8.6	21.3±2.7	15.1±1.5
	Week 5		Latency to vocalise (s)	23.6±5.1	24.0±2.8	27.8±4.0
***Feather pecking behaviour (pecks/20 min)***						
	Week 1		Gentle feather pecking	**24.4±1.9^a^**	**9.7±1.7^b^**	**16.3±4.1^c^**
	Week 5		Gentle feather pecking	70.6±27.4	74.8±17.1	41.9±9.7
	Week 10		Gentle feather pecking	**71.1±14.6^a^**	**23.8±5.2^b^**	**42.9±11.8^c^**
	Week 1		Severe feather pecking	4.0±1.0	1.7±0.5	2.1±0.9
	Week 5		Severe feather pecking	**15.4±5.8^x^**	**9.6±1.6^x^**	**4.0±1.6^y^**
	Week 10		Severe feather pecking	**7.3±1.9^a^**	**1.6±0.5^b^**	**2.2±0.8^c^**
***Feather damage scoring (min = 0, max = 2)***						
	Week 5		Average feather score	0.24±0.07	0.28±0.05	0.31±0.05
	Week 10		Average feather score	**0.29±0.08^a^**	**0.23±0.03^b^**	**0.22±0.05^b^**
	Week 15		Average feather score	0.23±0.04	0.14±0.02	0.13±0.02

Bold number with superscripts a,b,c indicate P-value of <0.05; bold numbers with superscripts x,y,z indicate P-value <0.1>0.05 (different superscript letters indicate pair-wise differences), ” –“ indicate non determined effect due to effects of an artifact of the system on the response variable.

### Genetic effects

GFP tended to be higher for DW than for ISA birds at one week of age (GFP_week1_: F_1,38_ = 3.69, P = 0.06 : DW: 16.8±3.2 pecks/20 min vs. ISA: 11.4±1.7 pecks/20 min). At five and ten weeks of age GFP did not differ between hybrids (GFP_week5_: F_1,44_ = 0.11, P = 0.73, GFP_week10_: F_1,45_ = 0.01, P = 0.94). SFP was not affected by hybrid at one or ten weeks of age (SFP_week1_: F_1,39_ = 0.00, P = 0.97: SFP_week10_: F_1,45_ = 1.16, P = 0.29). SFP at week 5 of age was affected by the interaction of hybrid with litter limitation, which will be explained further-on under litter effects. At ten weeks of age, but not at one or five weeks of age, DW birds kept a greater distance to the human observer than ISA birds (SPT_week1_: F_1,28_ = 0.77, P = 0.39; SPT_week5_: F_1,28_ = 0.09, P = 0.76; SPT_week10_ F_1,28_ = 12.15, P = 0.002: DW: 152.9±17.8 cm vs. ISA: 57.9±11.0 cm). Whole-blood serotonin (5-HT) was higher for ISA birds than for DW birds (F_1,44_ = 64.03, P < 0.001: DW: 60.8±1.26 nmol/ml vs. ISA: 88.6±2.54 nmol/ml). Plasma CORT was not affected by hybrid (F_1,44_ = 0.00, P = 0.96: DW: 1.85±0.06 ng/ml vs. ISA: 2.05± 0.15 ng/ml).

### Litter effects

**Figure 3:**
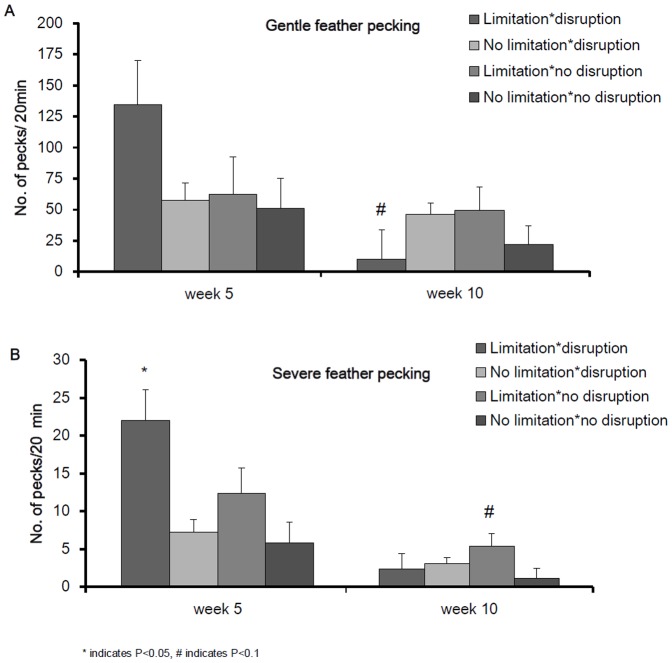
A. Gentle feather pecking at 5 and 10 weeks of age in relation to litter disruption and litter limitation B. Severe feather pecking at 5 and 10 weeks of age in relation to litter disruption and litter limitation.

**Figure 4:**
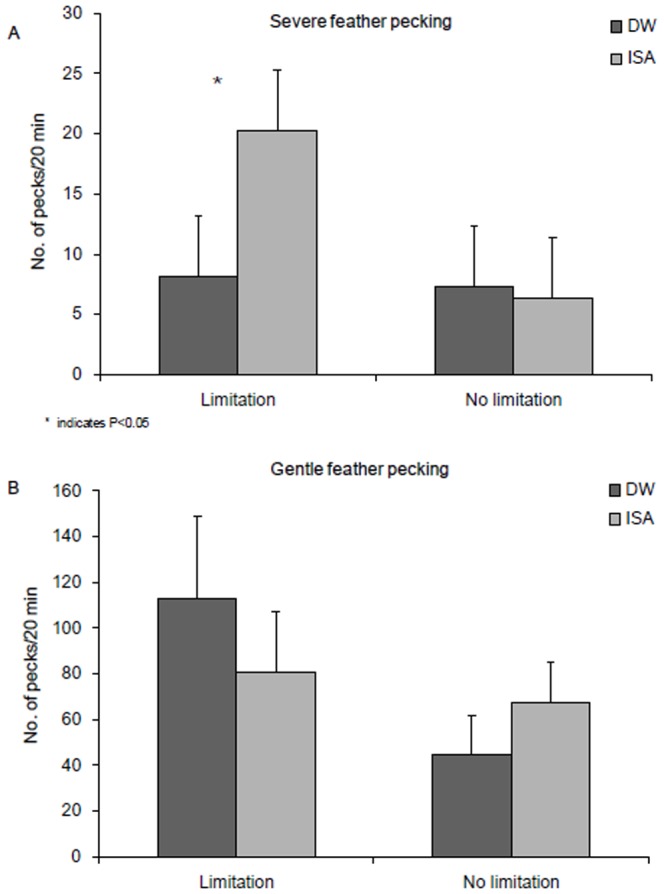
A. Severe feather pecking at 5 weeks of age in Dekalb White (DW) and ISA brown (ISA) birds in relation to litter limitation B. Gentle feather pecking at 5 weeks of age in Dekalb White (DW) and ISA brown (ISA) birds in relation to litter limitation.

**Figure 5:**
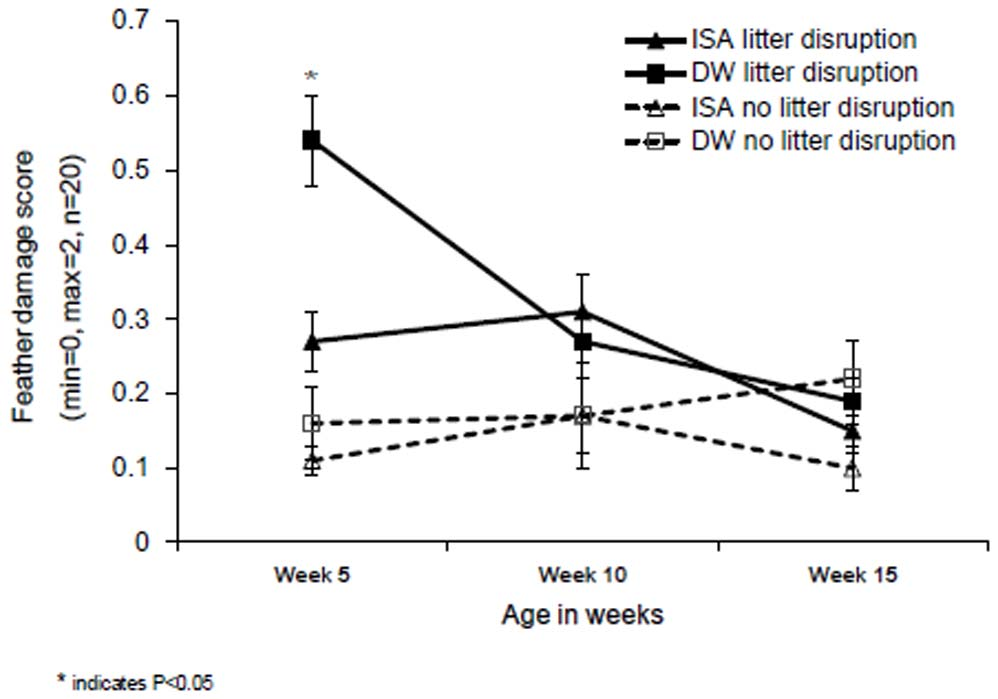
Feather damage score of Dekalb White (DW) and ISA brown (ISA) at 5, 10 and 15 weeks of age in relation to litter disruption.

**Figure 6:**
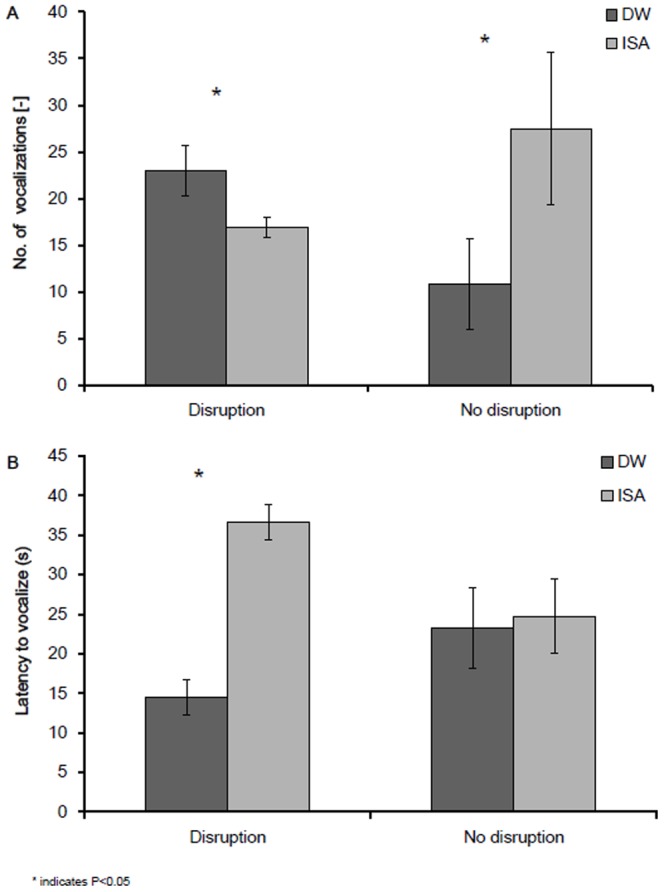
A. Number of vocalizations upon social isolation at 5 weeks of age in Dekalb White (DW) and ISA Brown (ISA) chicks in relation to litter disruption B. Latency to vocalize upon social isolation at 5 weeks of age in Dekalb White (DW) and ISA Brown (ISA) chicks in relation to litter disruption.

The combination of both litter disruption and litter limitation resulted in the highest levels of SFP at five weeks of age (litter disruption * litter limitation: F_1,43_ = 4.12, P = 0.05, Figure 3a) and a similar but non-significant trend for GFP at five weeks (litter disruption * litter limitation: F_1,44_ = 1.13, P = 0.30, Figure 3b). GFP and SFP at week 10 of age also tended to be affected by the interaction between limitation and disruption (litter limitation * litter disruption: GFP_week10_: F_1,45_ = 3.12, P = 0.08; SFP_week10:_ F_1,45_ = 3.32, P = 0.08, Figure 3a,b). Limitation of litter alone increased SFP at five weeks in the ISA hybrid but not in the DW hybrid (hybrid * limitation: F_1,43_ = 7.36, P = 0.01, see Figure 4a) while GFP did not differ between hybrids (hybrid * limitation: F_1,44_ = 0.04, P = 0.84, Figure 4b). Disruption of litter alone increased feather damage score at week 5 and 10 but not at 15 weeks of age (disruption: FS_week5_: F_1,45_ = 18.55, P = 0.002, FS_week10_ : F_1,45_ = 6.55, P = 0.02, FS_week15_: F_1,45_ = 0.48, P = 0.51, Table 2). These effects were most strong for the DW hybrid at five weeks of age (hybrid * disruption: FS_week5_: F_1,45_ = 4.21, P = 0.05, FS_week10_ : F_1,45_ = 0.34, P = 0.56, FS_week15_: F_1,45_ = 0.79, P = 0.35, Figure 5). Independent of hybrid, in flocks which experienced a litter disruption, birds tended to keep a greater distance to the human observer (litter disruption: F_1,44_ = 3.00, P = 0.09: disruption: 126.7±15.7 cm vs. no disruption: 63.9±17.0 cm) and tended to approach a novel object later (litter disruption: F_1,43_ = 3.78, P = 0.06, disruption: 31.1±5.0 s. vs. no disruption: 17.0±2.4 s.) in comparison to flocks that did not experience litter disruption. Whole-blood 5-HT was higher when litter was disrupted then when litter was not disrupted (litter disruption: F_1,44_ = 4.24, P = 0.05; disruption: 64.2±3.6 nmol/ml vs. no disruption: 57.0±3.5 nmol/ml). Plasma-corticosterone was not affected by litter supply (litter disruption: F_1,44_ = 0.49, P = 0.48, litter limitation: F_1,44_ = 0.18, P = 0.67). Disruption in access to litter affected the response to social isolation at five weeks differently between the hybrids; ISA birds that had a disruption in litter supply vocalized less than ISA birds that did not have a disruption in litter supply (hybrid * disruption: F_1,43_ = 4.08, P = 0.05) and had a longer latency to vocalize (hybrid * disruption: F_1,43_ = 3.63, P = 0.04) while the opposite was the case for the DW birds (Figure 6).

**Table 2:** Feather damage score at week five, ten and fifteen of age in relation to litter disruption.

	Disruption	
Feather damage score	Yes	No
week 5	0.45(0.06)^a^	0.18(0.05)^b^
week 10	0.29(0.06)^a^	0.15(0.06)^b^
week 15	0.16(0.03)	0.15(0.03)

Numbers with superscripts a,b indicate P-value of <0.05.

### Relations between anxiety and feather pecking

**Figure 7:**
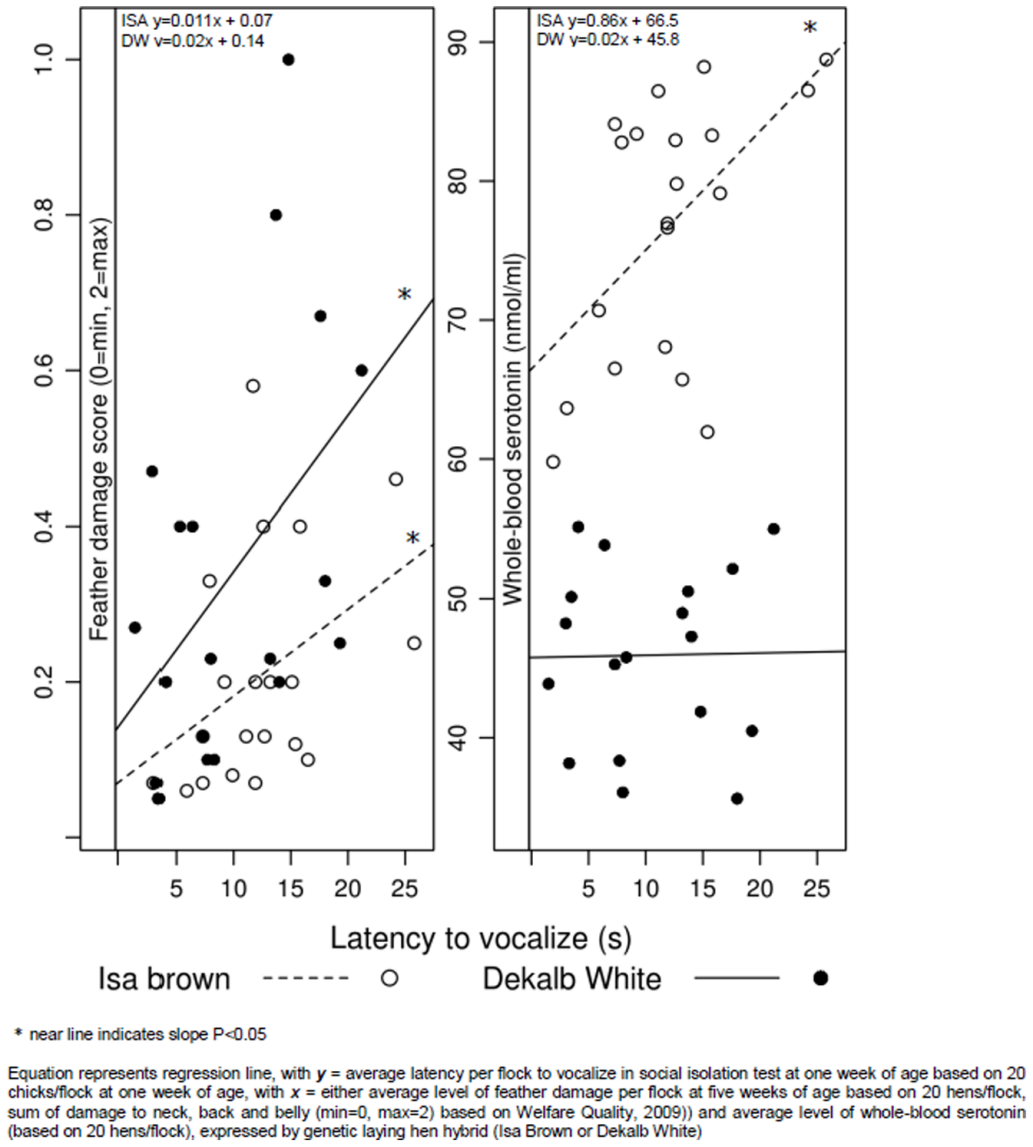
Average level of feather damage score at five weeks of age [left panel] and average level of whole-blood serotonin at 15 weeks of age [right panel] related to the average latency to vocalize in a social isolation at one week of age in flocks of Dekalb White (DW) and ISA Brown (ISA) laying hens.

For both hybrids, average feather damage score at five weeks was higher when the latency to vocalize in the social isolation test at one week of age was higher (*r* = 0.46, P < 0.003, Figure 7). In the ISA birds, whole-blood serotonin levels were higher if the latency to vocalize in the social isolation test at one week was higher (*r_ISA_* = 0.67, P < 0.001, Figure 7), but this was not significant in the DW birds (*r_DW_* = 0.22, P = 0.37). As 5-HT was higher for birds which experienced a litter disruption, we assessed the correlation within litter disruption groups within the ISA hybrid. For litter disruption the correlation between 5-HT and vocalizations at one week was positive (*r_limitation_* = 0.72, P = 0.02), while without litter disruption the correlation was not significant (*r_no limitation_* = 0.17, P = 0.70).

## Discussion

This is the first on-farm study in which maternal effects on the behavioral development of offspring are described for laying hens. We explored and examined which maternal and environmental effects act on the development of feather pecking (FP) from one until fifteen weeks of age in two hybrids: Dekalb White (DW) and ISA Brown (ISA). As FP is related to anxiety [30], [31], we also assessed this relationship under commercial conditions.

### Maternal effects

In the DW hybrid, high maternal plasma-corticosterone (CORT), whole-blood serotonin (5-HT) and feather damage were positively related to offsprings’ severe FP (SFP) at one week of age and offsprings’ vocalizations upon social isolation at one and five weeks of age. The latter are indicative of fearfulness and anxiety [55]–[57]. These results suggest that within the DW hybrid, maternal state can affect behavioural development of the offspring and thereby cause high fearfulness [55], [56] and SFP. These maternal effects may derive from high levels of stress (affecting CORT) and feather pecking in the maternal birds (affecting 5-HT and feather damage, for details see [24] ). Offspring of mothers with high CORT have repeatedly shown to have high levels of fearfulness (hens [7], quail [22], [58], [59]) and emotional reactivity (hens [15], [16], quail [21], [26], [60]). Altered deposition of nutrients and hormones in the egg may underlie the maternal effects we found (for review see [12]). High CORT of the mother, due to living in a stressful environment, can affect yolk-hormones such as testosterone [7], [21], [26], [60], [61], progesterone [22], [60] and oestrogens [15], [20] which can influence offspring behavior [12], [14]. Additionally, high maternal CORT has been related to low egg weight [23], [24] and chick weight post hatch [7], [16], [22]. It is known that ISA and DW birds with high maternal CORT induced by CORT implants differ in yolk-steroid levels and yolk-mass [23]. Offsprings’ fearfulness and SFP in our study may thus be influenced by egg hormone and nutrient content as affected by maternal physiology. In the present study these maternal effects depended on genotype. Breed-dependent differences in epigenetic programming (similar gene-expression patterns over generations and other non-genetic inheritable traits, see reviews on epigenetic studies in mice and chickens [62]–[65]) have also been identified as a putative mechanism of maternal effects [15]–[17]. These epigenetic changes may even be induced by altered egg-hormone content [15], [60], [66]. Differences in genetic and epigenetic inheritance between laying hen lines may be the reason why we only recorded maternal effects in the DW hybrid and not in the ISA hybrid.

### Environmental effects

#### Housing effects

In the open level system, chicks had a shorter latency to approach a novel object, but also had the highest gentle FP (GFP) at one and ten weeks of age, highest SFP at five (a tendency P<0.1) and ten weeks of age, and highest feather damage at ten weeks of age compared to chicks housed in a closed or partly-open aviary system. Although GFP and SFP originate from different behavioral needs [28], [42] and involvement of different genes [67] and gene-expression patterns [68], one does not necessarily lead to the other [31], [42], [69], but the co-existence of both may result in feather damage. In the open level system chicks are placed together with thousands of other individuals inside a large area from day one. In both aviary systems group size is substantially smaller than in the level system as the (partially) closed walls of the aviary system limit the space nor group size to extent to over hundreds. Effects of system are therefore likely to partly be group size related. Social transmission of behavior [70], [71], such as the approach of novel objects and FP, may have occurred more readily in a large group [72] as there are more birds from which to copy and synchronize behavior. Previous studies suggest that FP is socially transmitted within a group (SFP [34], [73], [74], GFP [74]–[76]). In the closed aviary system we recorded a peak in GFP at five weeks of age. GFP seems to stem from social exploration [28], [76] and presumably underlies this result. Birds are mixed at around four to five weeks, and this may elicit social exploration, which presumably would have already occurred in the other systems. These results indicate that housing system (possibly related to differences in group size which affect social exploration and social transmission) influences the development of FP and feather damage on-farm.

### Litter effects

Litter disruption (taking away foraging substrate for a period of 7–10 days) and litter limitation (limited supplementation in the form of remnant of chicken paper) had a substantial effect on FP and fear responses (Table 3). Especially at five weeks of age, disruption of litter led to high SFP, GFP and feather damage. During litter disruption, three factors are at play: 1) disturbances by the farmer, who takes out cardboard paper, handles and mixes birds, 2) removal of cardboard paper and thus removal of foraging material, and 3) disrupted uptake of fibers or excretions from cardboard paper.

**Table 3:** Effects of litter supply (litter disruption and litter limitation) on feather pecking, fearfulness and whole-blood serotonin in laying hens.

Effects of litter supply	Severe feather pecking	Gentle feather pecking	Feather damage	Stationary person test	Novel object test	Social isolation test	Whole-blood 5-HT	Correlations
Disruption			↑ week 5** ↑ week 10^#^	↑ week 10^#^	↑ week 10^#^	↑ week 5 (ISA)*	↑ week 15*	5-HT at 15 weeks & social isolation test at 5 weeks ^r = 0.67^**
Limitation	↑ week 5 (ISA)*	↑ week 5^*^						
Disruption* limitation	↑ week 5^*^ ∼ week 10^#^	↑ week 5^NS^						
**Effects displayed in:**	*Figure 3*	*Figure 3*	*Figure 5*	*text*	*text*	*Figure 6*	*text*	*Figure 7*

↑increase due to described effect, ∼ effects dependent on interaction, NS: not significant, ** indicates effects P<0.01, * indicates effects P<0.05, # indicates effects P<0.1, (ISA) effects only valid for ISA hybrid, *r = * correlation coefficient, week  =  age of hens in weeks

The first factor, disturbance by the farmer, may elicit stress related to fear of humans, as indicated by the greater distance to the human observer in flocks in which access to litter was disrupted. Additionally, absence of litter may induce frustration which can result in SFP [77]. The act of SFP itself (pecking and pulling feathers) causes distress in the victims i.e. withdrawal, escape attempts, vocalizations [42] and can lead to disturbances in the flock [78], [79]. Taken together, litter disruption can, either directly or indirectly via SFP, increase a flock’s fear level.

The second factor, removal of foraging substrate, has most probably the largest influence on the occurrence of FP. FP is considered redirected foraging pecking [80], and increases when foraging material is limited [43], [81], especially at an early age [48], [49]. As said before, lack of foraging material can induce frustration when the need to forage is thwarted [77] and results in SFP which in turn can lead to feather damage, as shown in this study. On top of litter disruption, a subsequent limitation of litter brought an additive effect in the development of SFP. At any given time, foraging material is important in prevention of SFP [81]. Feather damage seemed to reduce when birds age, irrespective of litter supply. This may be influenced by the molting periods around 10 weeks of age [82], making loose feathers available for ingestion from the floor [83]. Feather pecking during early rearing, as affected by litter supply, may however still yield a risk of later outbreaks of feather damage during lay [42], [84].

The third factor, lack of uptake of fibers or excretions, probably affected the level of 5-HT, as our study shows increased whole blood 5-HT levels in flocks with litter disruption. Litter (often wood shavings, alfalfa or remaining cardboard paper) contains fibers, excretions and feather particles. Uptake of these large particles can stimulate gut motility [85], alter gut micro biota [86], [87] and activate immunity in various ways [88]. Particularly feather eating, which is linked with FP [83], [90], [91] has been associated with increased gut motility [92]. The enterochromaffin cells in the gut contain 5-HT which are released upon stimulation of the intestinal tract [89]. As a result of a temporary lack of litter birds may have a strong need to forage, possible enhancing feather and litter uptake afterwards as over-compensation [83] which altogether affects 5-HT release. Our study shows that whole-blood 5-HT can be influenced by litter disruption.

In the ISA hybrid, especially under disruption of litter, a positive correlation between fear-response at one week of age and 5-HT at fifteen weeks of age was detected. In a previous study, in Rhode Island Red birds (RIR), one of the founder lines of ISA, the correlation between fear responses and brain 5-HT was also dependent on the environment. RIR birds mixed with birds of another line showed a negative correlation between fear and 5-HT while RIR birds which we kept in non-mixed groups showed a positive correlation [38]. The positive correlation between fear-responses and 5-HT under litter disruption in our study could be influenced by effects of mixing and substrate intake but probably also by the high levels of SFP occurring under litter disruption. 5-HT activity has been suggested to relate to the development of SFP [42] (brain 5-HT young [90] and adult birds [91], peripheral 5-HT [32], [33] and both brain and peripheral 5-HT [38]). Both brain and peripheral 5-HT have also been associated with fearfulness [32], [33], [92], and, in our study, peripheral 5-HT was influenced by litter disruption.

Within the ISA hybrid, disruption of litter also caused higher anxiety in the social isolation test at five weeks of age. ISA birds appear to be more strongly affected by their (social) environment than DW birds [38]. In comparison to other hybrids, birds from a brown origin (in the PS [24] and founder lines [36]) repeatedly show higher fear in response to social isolation [93]–[95] and novel items in their home environment. ISA birds are also more affected by social factors such as group size [24] and mixing [34], [37] than DW birds. Taken together with other studies, it appears that ISA birds are more strongly affected by their (social) environment in comparison to DW birds who are more sensitive to maternal effects.

### Fear and feather pecking

For both hybrids we found that latency to vocalize during social isolation at one week of age was related to feather damage at five weeks of age, which complements the relationship between anxiety traits in social isolation tests and FP [30], [31]. This may also explain why we still see FP under optimal conditions with regard to litter. In DW birds, fear of humans was higher than in ISA birds, which was similar to the study of the PS [24]. DW birds are more easily frightened by exposure to humans [34] as indicated by higher fear-responses and plasma-CORT after human handling [35], [36]. DW birds also have relatively low levels of whole-blood 5-HT compared to ISA birds (shown in this study, in the PS [24] and founder lines [38]), which may represent a risk in the development of FP [42]. In addition, the maternal effects on fearfulness in early life may predispose DW birds to develop SFP. The predisposition to develop FP may thus stem from different origins depending on genotype.

## Conclusion

This study shows for the first time that maternal effects in commercial laying hens play an important role in early life behavioral development of their offspring. Our study indicates that maternal stress can create a risk for the development of anxiety and maladaptive behavior such as feather pecking (FP) in laying hens. These maternal effects depend on genotype, with birds from a White Leghorn origin being sensitive. Litter availability is of utmost importance for laying hens, and reduced the risk of FP, especially for birds from a Rhode Island Red origin who also become more anxious and fearful as a result of disruption in litter supply. These results provide new knowledge that is important for preventing the development of anxiety and FP in laying hens.

## References

[1] Lumey LH, Stein AD, Kahn HS, van der Pal-de Bruin KM, Blauw GJ, et al (2007) Cohort Profile: The Dutch Hunger Winter Families study. International Journal of Epidemiology 36: 1196–1204. 17591638 10.1093/ije/dym126

[2] Viltart O, Vanbesien-Mailliot CCA (2007) Impact of prenatal stress on neuroendocrine programming. The Scientific World Journal 7: 1493–1537. 17767365 10.1100/tsw.2007.204 PMC5901338

[3] Champagne FA, Rissman EF (2011) Behavioral epigenetics: A new frontier in the study of hormones and behavior. Hormones and Behavior 59: 277–278. 21419246 10.1016/j.yhbeh.2011.02.011

[4] Weaver ICG, Cervoni N, Champagne FA, D'Alessio AC, Sharma S, et al (2004) Epigenetic programming by maternal behavior. Nature Neuroscience 7: 847–854. 15220929 10.1038/nn1276

[5] Eriksen MS, Faerevik G, Kittilsen S, McCormick MI, Damsgard B, et al (2011) Stressed mothers - troubled offspring: a study of behavioural maternal effects in farmed Salmo salar. Journal of Fish Biology 79: 575–586. 21884101 10.1111/j.1095-8649.2011.03036.x

[6] Groothuis TGG, Muller W, Von Engelhardt N, Carere C, Eising C (2005) Maternal hormones as a tool to adjust offspring phenotype in avian species. Neuroscience and Biobehavioral Reviews 29: 329–352. 15811503 10.1016/j.neubiorev.2004.12.002

[7] Janczak AM, Torjesen P, Palme R, Bakken M (2007) Effects of stress in hens on the behaviour of their offspring. Applied Animal Behaviour Science 107: 66–77.

[8] Gudsnuk KMA, Champagne FA (2011) Epigenetic Effects of Early Developmental Experiences. Clinics in Perinatology 38: 703–+. 10.1016/j.clp.2011.08.005 22107899

[9] Charil A, Laplante DP, Vaillancourt C, King S (2010) Prenatal stress and brain development. Brain Research Reviews 65: 56–79. 20550950 10.1016/j.brainresrev.2010.06.002

[10] Brunton PJ, Russell JA (2010) Prenatal Social Stress in the Rat Programmes Neuroendocrine and Behavioural Responses to Stress in the Adult Offspring: Sex-Specific Effects. Journal of Neuroendocrinology 22: 258–271. 20136688 10.1111/j.1365-2826.2010.01969.x

[11] Rutherford KMD, Donald RD, Arnottt G, Rooke JA, Dixon L, et al (2012) Farm animal welfare: assessing risks attributable to the prenatal environment. Animal Welfare 21: 419–429.

[12] Henriksen R, Rettenbacher S, Groothuis TGG (2011) Prenatal stress in birds: Pathways, effects, function and perspectives. Neuroscience and Biobehavioral Reviews 35: 1484–1501. 21536067 10.1016/j.neubiorev.2011.04.010

[13] Richard-Yris MA, Michel N, Bertin A (2005) Nongenomic inheritance of emotional reactivity in Japanese quail. Developmental Psychobiology 46: 1–12. 15690384 10.1002/dev.20040

[14] Groothuis TGG, Schwabl H (2008) Hormone-mediated maternal effects in birds: mechanisms matter but what do we know of them? Philosophical Transactions of the Royal Society B-Biological Sciences 363: 1647–1661. 10.1098/rstb.2007.0007 PMC2606725 18048291

[15] Natt D, Lindqvist N, Stranneheim H, Lundeberg J, Torjesen PA, et al (2009) Inheritance of Acquired Behaviour Adaptations and Brain Gene Expression in Chickens. PLoS One 4: e6405. 19636381 10.1371/journal.pone.0006405 PMC2713434

[16] Goerlich VC, Nätt D, Elfwing M, Macdonald B, Jensen P (2012) Transgenerational effects of early experience on behavioral, hormonal and gene expression responses to acute stress in the precocial chicken. Hormones and Behavior 61: 711–718. 22465454 10.1016/j.yhbeh.2012.03.006

[17] Lindqvist C, Janczak AM, Natt D, Baranowska I, Lindqvist N, et al (2007) Transmission of stress-induced learning impairment and associated brain gene expression from parents to offspring in chickens. PLoS One 2: e364. 17426812 10.1371/journal.pone.0000364 PMC1838921

[18] Bateson P (2007) Developmental plasticity and evolutionary biology. Journal of Nutrition 137: 1060–1062. 17374677 10.1093/jn/137.4.1060

[19] Gluckman PD, Hanson MA, Spencer HG (2005) Predictive adaptive responses and human evolution. Trends in Ecology & Evolution 20: 527–533. 16701430 10.1016/j.tree.2005.08.001

[20] Janczak AM, Torjesen P, Rettenbacher S (2009) Environmental effects on steroid hormone concentrations in laying hens' eggs. Acta Agriculturae Scandinavica Section a-Animal Science 59: 80–84.

[21] Guibert F, Richard-Yris M-A, Lumineau S, Kotrschal K, Bertin A, et al (2011) Unpredictable mild stressors on laying females influence the composition of Japanese quail eggs and offspring's phenotype. Applied Animal Behaviour Science 132: 51–60.

[22] Bertin A, Richard-Yris M-A, Houdelier C, Lumineau S, Moestl E, et al (2008) Habituation to humans affects yolk steroid levels and offspring phenotype in quail. Hormones and Behavior 54: 396–402. 18572170 10.1016/j.yhbeh.2008.04.012

[23] Henriksen R, Groothuis TG, Rettenbacher S (2011) Elevated Plasma Corticosterone Decreases Yolk Testosterone and Progesterone in Chickens: Linking Maternal Stress and Hormone-Mediated Maternal Effects. Plos One 6. 10.1371/journal.pone.0023824 PMC3160319 21886826

[24] de Haas EN, Kemp B, Bolhuis JE, Groothuis T, Rodenburg TB (2013) Fear, stress, and feather pecking in commercial white and brown laying hen parent-stock flocks and their relationships with production parameters. Poultry Science 92: 2259–2269. 10.3382/ps.2012-02996 23960107

[25] Janczak AM, Heikkila M, Valros A, Torjesen P, Andersen IL, et al (2007) Effects of embryonic corticosterone exposure and post-hatch handling on tonic immobility and willingness to compete in chicks. Applied Animal Behaviour Science 107: 275–286.

[26] Guibert F, Richard-Yris M-A, Lumineau S, Kotrschal K, Guemene D, et al.. (2010) Social Instability in Laying Quail: Consequences on Yolk Steroids and Offspring's Phenotype. Plos One 5. 10.1371/journal.pone.0014069 PMC2989911 21124926

[27] Davis KA, Schmidt JB, Doescher RM, Satterlee DG (2008) Fear responses of offspring from divergent quail stress response line hens treated with corticosterone during egg formation. Poultry Science 87: 1303–1313. 10.3382/ps.2008-00083 18577609

[28] Savory CJ (1995) Feather pecking and cannibalism. World's Poultry Science Journal 51: 215–219.

[29] Nicol CJ, Bestman M, Gilani A-M, De Haas EN, De Jong IC, et al (2013) The prevention and control of feather pecking: application to commercial systems. World's Poultry Science Journal 69: 775–788.

[30] Jones RB, Blokhuis HJ, Beuving G (1995) Open-field and tonic immobility responses in domestic chicks of two genetic lines differing in their propensity to feather peck. British Poultry Science 36: 525–530. 10.1080/00071669508417798 8590085

[31] Rodenburg TB, Buitenhuis AJ, Ask B, Uitdehaag KA, Koene P, et al (2004) Genetic and phenotypic correlations between feather pecking and open-field response in laying hens at two different ages. Behavior Genetics 34: 407–415. 15082938 10.1023/B:BEGE.0000023646.46940.2d

[32] Bolhuis JE, Ellen ED, Van Reenen CG, De Groot J, Ten Napel J, et al (2009) Effects of genetic group selection against mortality on behaviour and peripheral serotonin in domestic laying hens with trimmed and intact beaks. Physiology & Behavior 97: 470–475. 19341749 10.1016/j.physbeh.2009.03.021

[33] Rodenburg TB, Bolhuis JE, Koopmanschap RE, Ellen ED, Decuypere E (2009) Maternal care and selection for low mortality affect post-stress corticosterone and peripheral serotonin in laying hens. Physiology & Behavior 98: 519–523. 19699216 10.1016/j.physbeh.2009.08.006

[34] Uitdehaag KA, Rodenburg TB, Bolhuis JE, Decuypere E, Komen H (2009) Mixed housing of different genetic lines of laying hens negatively affects feather pecking and fear related behaviour. Applied Animal Behaviour Science 116: 58–66.

[35] Fraisse F, Cockrem JF (2006) Corticosterone and fear behaviour in white and brown caged laying hens. British Poultry Science 47: 110–119. 10.1080/00071660600610534 16641020

[36] Uitdehaag K, Kornen H, Rodenburg TB, Kemp B, van Arendonk J (2008) The novel object test as predictor of feather damage in cage-housed Rhode Island Red and White Leghorn laying hens. Applied Animal Behaviour Science 109: 292–305.

[37] Uitdehaag KA, Rodenburg TB, van Hierden YM, Bolhuis JE, Toscano MJ, et al (2008) Effects of mixed housing of birds from two genetic lines of laying hens on open field and manual restraint responses. Behavioural Processes 79: 13–18. 18511218 10.1016/j.beproc.2008.04.004

[38] Uitdehaag KA, Rodenburg TB, Van Reenen CG, Koopmanschap RE, Reilingh GD, et al (2011) Effects of genetic origin and social environment on behavioral response to manual restraint and monoamine functioning in laying hens. Poultry Science 90: 1629–1636. 10.3382/ps.2010-01292 21753196

[39] Janczak AM, Braastad BO, Bakken M (2006) Behavioural effects of embryonic exposure to corticosterone in chickens. Applied Animal Behaviour Science 96: 69–82.

[40] Rodenburg TB, Komen H, Ellen ED, Uitdehaag KA, van Arendonk JAM (2008) Selection method and early-life history affect behavioural development, feather pecking and cannibalism in laying hens: A review. Applied Animal Behaviour Science 110: 217–228.

[41] Rogers LJ (1995) Behavioural transitions in early posthatching life. The development of brain and behaviour in the chicken. Wallingford, United Kingdom: CAB International. pp. 157–183.

[42] Rodenburg TB, van Krimpen MM, de Jong IC, de Haas EN, Kops MS, et al (2013) The prevention and control of feather pecking in laying hens: identifying the underlying principles. Worlds Poultry Science Journal 69: 361–373.

[43] Gilani AM, Knowles TG, Nicol CJ (2012) The effect of dark brooders on feather pecking on commercial farms. Applied Animal Behaviour Science 142: 42–50.

[44] Bilcík B, Keeling LJ (2000) Relationship between feather pecking and ground pecking in laying hens and the effect of group size. Applied Animal Behaviour Science 68: 55–66. 10771315 10.1016/s0168-1591(00)00089-7

[45] Kjaer JB (2004) Effects of stocking density and group size on the condition of the skin and feathers of pheasant chicks. The Veterinairy Record 154: 556–558. 10.1136/vr.154.18.556 15144000

[46] Rodenburg TB, Koene P (2007) The impact of group size on damaging behaviours, aggression, fear and stress in farm animals. Applied Animal Behaviour Science 103: 205–214.

[47] Zimmerman PH, Lindberg AC, Pope SJ, Glen E, Bolhuis JE, et al (2006) The effect of stocking density, flock size and modified management on laying hen behaviour and welfare in a non-cage system. Applied Animal Behaviour Science 101: 111–124.

[48] de Jong IC, Reuvekamp BFJ, Gunnink H (2013) Can substrate in early rearing prevent feather pecking in adult laying hens? Animal Welfare 22: 305–314.

[49] Huber-Eicher B, Wechsler B (1998) The effect of quality and availability of foraging materials on feather pecking in laying hen chicks. Animal Behaviour 55: 861–873. 9632473 10.1006/anbe.1997.0715

[50] HuberEicher B, Wechsler B (1997) Feather pecking in domestic chicks: its relation to dustbathing and foraging. Animal Behaviour 54: 757–768. 9344430 10.1006/anbe.1996.0506

[51] Welfare Quality (2009) Welfare Quality® assessment protocol for poultry (broilers, laying hens). Welfare Quality® Consortium, Lelystad, the Netherlands.

[52] Collias NE (1987) The vocal repertoire of the Red Junglefowl: a spectrographic classification and the code of communication. The Condor 89: 510–524.

[53] Sufka KJ, Hughes RA (1991) Differential effects of handling on isolation-induced vocalizations, hypoalgesia and hyperthermia in domestic fowl. Physiology & Behavior 50: 129–133. 1946704 10.1016/0031-9384(91)90508-l

[54] Warnick JE, Huang CJ, Acevedo EO, Sufka KJ (2009) Modelling the anxiety-depression continuum in chicks. Journal of Psychopharmacology 23: 143–156. 18515452 10.1177/0269881108089805

[55] Suarez SD, Gallup GG (1983) Social reinstatement and Open-Field testing in chickens. Animal Learning & Behavior 11: 119–126.

[56] Gallup GG, Suarez SD (1980) An ethological analysis of open-field behaviour in chickens. Animal Behaviour 28: 368–378.

[57] Faure JM, Jones RB, Bessei W (1983) Fear and social motivation as factors in open-field behaviour of the domestic chick. Biology of Behaviour 8: 103–116.

[58] Houdelier C, Lumineau S, Bertin A, Guibert F, De Margerie E, et al.. (2011) Development of Fearfulness in Birds: Genetic Factors Modulate Non-Genetic Maternal Influences. Plos One 6. 10.1371/journal.pone.0014604 PMC3029269 21298038

[59] Bertin A, Richard-Yris MA, Houdelier C, Richard S, Lumineau S, et al (2009) Divergent selection for inherent fearfulness leads to divergent yolk steroid levels in quail. Behaviour 146: 757–770.

[60] Guibert F, Lumineau S, Kotrschal K, Mostl E, Richard-Yris MA, et al.. (2013) Trans-generational effects of prenatal stress in quail. Proceedings of the Royal Society B-Biological Sciences 280. 10.1098/rspb.2012.2368 PMC3574342 23256192

[61] Schwabl H (1997) The contents of maternal testosterone in house sparrow Passer domesticus eggs vary with breeding conditions. Naturwissenschaften 84: 406–408. 9353760 10.1007/s001140050418

[62] Champagne FA (2010) Early Adversity and Developmental Outcomes: Interaction Between Genetics, Epigenetics, and Social Experiences Across the Life Span. Perspectives on Psychological Science 5: 564–574. 26162197 10.1177/1745691610383494

[63] Champagne FA (2012) Interplay Between Social Experiences and the Genome: Epigenetic Consequences for Behavior. In: Sokolowski MB, Goodwin SF, editors. Gene-Environment Interplay. pp. 33–57. 10.1016/B978-0-12-387687-4.00002-7 22902125

[64] Curley JP, Mashoodh R, Champagne FA (2011) Epigenetics and the origins of paternal ffects. Hormones and Behavior 59: 306–314. 20620140 10.1016/j.yhbeh.2010.06.018 PMC2975825

[65] Berghof T, Parmentier H, Lammers A (2013) Transgenerational epigenetic effects on innate immunity in broilers: An underestimated field to be explored? Poultry Science 92: 2904–2913. 10.3382/ps.2013-03177 24135594

[66] Ho DH, Burggren WW (2010) Epigenetics and transgenerational transfer: a physiological perspective. Journal of Experimental Biology 213: 3–16. 20008356 10.1242/jeb.019752

[67] Buitenhuis AJ, Rodenburg TB, Wissink PH, Visscher J, Koene P, et al (2004) Genetic and phenotypic correlations between feather pecking behavior, stress response, immune response, and egg quality traits in laying hens. Poultry Science 83: 1077–1082. 10.1093/ps/83.7.1077 15285495

[68] Hughes AL, Buitenhuis AJ (2010) Reduced variance of gene expression at numerous loci in a population of chickens selected for high feather pecking. Poultry Science 89: 1858–1869. 10.3382/ps.2010-00827 20709970

[69] Newberry RC, Keeling LJ, Estevez I, Bilcik B (2007) Behaviour when young as a predictor of severe feather pecking in adult laying hens: The redirected foraging hypothesis revisited. Applied Animal Behaviour Science 107: 262–274.

[70] Nicol CJ (1995) The social transmission of information and behavior. Applied Animal Behaviour Science 44: 79–98.

[71] Tolman CW (1964) Social Facilitation Of Feeding Behaviour In Domestic Chick. Animal Behaviour 12: 245–251. 10.1016/0003-3472(65)90111-9 5882808

[72] Croney CC, Newberry RC (2007) Group size and cognitive processes. Applied Animal Behaviour Science 103: 215–228.

[73] Zeltner E, Klein T, Huber-Eicher B (2000) Is there social transmission of feather pecking in groups of laying hen chicks? Animal Behaviour 60: 211–216. 10973723 10.1006/anbe.2000.1453

[74] McAdie TM, Keeling LJ (2002) The social transmission of feather pecking in laying hens: effects of environment and age. Applied Animal Behaviour Science 75: 147–159.

[75] McAdie TM, Keeling LJ (2000) Effect of manipulating feathers of laying hens on the incidence of feather pecking and cannibalism. Applied Animal Behaviour Science 68: 215–229. 10804267 10.1016/s0168-1591(00)00107-6

[76] Riedstra B, Groothuis TGG (2002) Early feather pecking as a form of social exploration: the effect of group stability on feather pecking and tonic immobility in domestic chicks. Applied Animal Behaviour Science 77: 127–138.

[77] Rodenburg TB, Koene P, Spruijt BM (2004) Reaction to frustration in high and low feather pecking lines of laying hens from commercial or semi-natural rearing conditions. Behavioural Processes 65: 179–188. 15222965 10.1016/j.beproc.2003.09.003

[78] Bright A (2008) Vocalisations and acoustic parameters of flock noise from feather pecking and non-feather pecking laying flocks. British Poultry Science 49: 241 – 249. 10.1080/00071660802094172 18568747

[79] Koene P, Zimmerman P, Bokkers E, Rodenburg B (2001) Vocalisation due to frustration in layer and broiler chickens. In: Oester H, Wyss C, editors. pp. 95–100.

[80] Blokhuis HJ (1986) Feather-pecking in poultry: its relation with ground-pecking. Applied Animal Behaviour Science 16: 63–67.

[81] Nicol CJ, Lindberg AC, Phillips AJ, Pope SJ, Wilkins LJ, et al (2001) Influence of prior exposure to wood shavings on feather pecking, dustbathing and foraging in adult laying hens. Applied Animal Behaviour Science 73: 141–155. 11358611 10.1016/s0168-1591(01)00126-5

[82] Savory CJ, Mann JS (1997) Behavioural development in groups of pen-housed pullets in relation to genetic strain, age and food form. British Poultry Science 38: 38–47. 10.1080/00071669708417938 9088611

[83] Harlander-Matauschek A, Benda I, Lavetti C, Djukic M, Bessei W (2007) The relative preferences for wood shavings or feathers in high and low feather pecking birds. Applied Animal Behaviour Science 107: 78–87.

[84] Bright A (2009) Time course of plumage damage in commercial layers. Veterinary Record 164: 334–335. 19287030 10.1136/vr.164.11.334

[85] Amerah AM, Ravindran V, Lentle RG, Thomas DG (2007) Feed particle size: Implications on the digestion and performance of poultry. Worlds Poultry Science Journal 63: 439–455.

[86] Meyer B, Bessei AW, Vahjen W, Zentek J, Harlander-Matauschek A (2012) Dietary inclusion of feathers affects intestinal microbiota and microbial metabolites in growing Leghorn-type chickens. Poultry Science 91: 1506–1513. 10.3382/ps.2011-01786 22700493

[87] Meyer B, Zentek J, Harlander-Matauschek A (2013) Differences in intestinal microbial metabolites in laying hens with high and low levels of repetitive feather-pecking behavior. Physiology & Behavior 110: 96–101. 23313560 10.1016/j.physbeh.2012.12.017

[88] Mossner R, Lesch KP (1998) Role of serotonin in the immune system and in neuroimmune interactions. Brain Behavior and Immunity 12: 249–271. 10.1006/brbi.1998.0532 10080856

[89] Ebert-Zavos E, Horvat-Gordon M, Taylor A, Bartell PA (2013) Biological Clocks in the Duodenum and the Diurnal Regulation of Duodenal and Plasma Serotonin. Plos One 8. 10.1371/journal.pone.0058477 PMC3667830 23737937

[90] van Hierden YM, de Boer SF, Koolhaas JM, Korte SM (2004) The Control of Feather Pecking by Serotonin. Behavioral Neuroscience 118: 575–583. 15174935 10.1037/0735-7044.118.3.575

[91] Kops MS, de Haas EN, Rodenburg TB, Ellen ED, Korte-Bouws GAH, et al (2013) Effects of feather pecking phenotype (severe feather peckers, victims and non-peckers) on serotonergic and dopaminergic activity in four brain areas of laying hens (Gallus gallus domesticus). Physiology and Behavior 120: 77–82. 23911692 10.1016/j.physbeh.2013.07.007

[92] Kops MS, de Haas EN, Rodenburg TB, Ellen ED, Korte-Bouws GAH, et al (2013) Selection for low mortality in laying hens affects catecholamine levels in the arcopallium, a brain area involved in fear and motor regulation. Behavioural Brain Research 257: 54–61. 24076385 10.1016/j.bbr.2013.09.035

[93] Ghareeb K, Niebuhr K, Awad WA, Waiblinger S, Troxler J (2008) Stability of fear and sociality in two strains of laying hens. British Poultry Science 49: 502 – 508. 10.1080/00071660802290390 18836895

[94] Hocking PM, Channing CE, Waddington D, Jones RB (2001) Age-related changes in fear, sociality and pecking behaviours in two strains of laying hen. British Poultry Science 42: 414–423. 10.1080/00071660120070686 11572615

[95] Ghareeb K, Awad WA, Niebuhr K, Böhm J, Troxler J (2008) Individual differences in fear and social reinstatement behaviours in laying hens. International Journal of Poultry Science 7: 843–851.

